# Increased Expression of ATG10 in Colorectal Cancer Is Associated with Lymphovascular Invasion and Lymph Node Metastasis

**DOI:** 10.1371/journal.pone.0052705

**Published:** 2012-12-20

**Authors:** Yoon Kyung Jo, Seung Cheol Kim, In Ja Park, So Jung Park, Dong-Hoon Jin, Seung-Woo Hong, Dong-Hyung Cho, Jin Cheon Kim

**Affiliations:** 1 Graduate School of East-West Medical Science, Kyung Hee University, Gyeonggi-do, Korea; 2 Institute for Innovative Cancer Research, Asan Medical Center, Seoul, Korea; 3 Department of Surgery, University of Ulsan College of Medicine and Asan Medical Center, Seoul, Korea; 4 Department of Oncology, University of Ulsan College of Medicine and Asan Medical Center, Seoul, Korea; Klinikum rechts der Isar der TU München, Germany

## Abstract

**Background:**

Autophagy has paradoxical and complex functions in cancer development, and autophagy-related genes (ATG) are key regulators in autophagy. Until now, more than 30 different ATG proteins have been identified in yeast, and their mammalian counterparts also have been reported. Although the roles of a few ATG proteins in cancer have been characterized, the role of ATG10 is almost completely unknown.

**Methodology/Principal Findings:**

To investigate the clinicopathological role of ATG10 in colorectal cancer, we analyzed ATG10 expression in colorectal cancer tissues and cell lines. Protein expression analysis showed that ATG10 is highly increased in colorectal cancer (tissue - 18/37 cases, 48%; cell line –8/12 cell lines, 66%). Immunohistochemical analysis with clinicopathological features indicated a strong association of the up-regulation of ATG10 with tumor lymph node metastasis (p = 0.005) and invasion (p<0.001). Moreover, both 5-year disease free survival and overall survival rates of patients bearing tumors that did not express ATG10 were significantly higher than those of patients bearing ATG10-expressing tumors (p = 0.012).

**Conclusion/Significance:**

Increased expression of ATG10 in colorectal cancer is associated with lymphovascular invasion and lymph node metastasis indicating that ATG10 may be a potential prognostic maker in colorectal cancer.

## Introduction

The ubiquitin/26S proteasome system is one of the major pathways regulating protein turnover in cells. Autophagy is the mechanism largely responsible for the removal of long-lived proteins and bulk turnover of cytosolic components. Autophagosomes are double-membrane vesicles that engulf target substrates and fuse with lysosomes to produce autolysosomes [1]. Autophagosome formation is regulated by a family of evolutionally conserved autophagy-related gene (ATG) proteins [2], [3].

Ubiquitin (Ub) conjugation is a well-coordinated event that requires E1, E2, and E3 enzymes [4]. Two protein conjugation systems, which are similar to those involved in protein ubiquitylation, are required for autophagic vesicles [3]. ATG7 acts as an E1-like activating enzyme, binding to ATG8/LC3 or ATG12. Activated ATG8 or ATG12 is then transferred to the E2-like conjugation enzymes, ATG3 and ATG10. Finally, the ATG8-phosphatidylethanolamine (PE) and ATG12-ATG5 conjugates are formed. The ATG12-ATG5 conjugate forms a complex with ATG16 to act as an E3-like enzyme for the ATG8-PE conjugate, which binds to the autophagosome membrane through a lipidation reaction [2]. Mutations in the binding sites of ATG7 and ATG10 prevent formation of the ATG12-ATG5 conjugate [5]. Because autophagy is involved in many cellular processes and homeostasis, deregulation of this system appears to play a role in many human pathophysiologic conditions such as neurodegenerative disease, type 2 diabetes, infectious disease, innate immune disease, as well as cancer [6].

Like other diseases, the role of autophagy in cancer is quite complex. Recent studies have demonstrated that down-regulation of ATG genes (or their regulators) directly or indirectly accelerate tumor development [7], [8]. Moreover, decreased autophagy enhances necrosis-dependent inflammation, further promoting tumor development [9]. However, other studies have suggested that increased autophagy can also assist tumor development. Indeed, autophagy promotes cell survival following stresses by regulating metabolic homeostasis. Tumor cells have to survive in hypoxia and dissociation from surrounding cells. Thus, autophagy could promote tumorigenesis and metastasis by increasing tumor cell survival [7], [10]. To date, more than 30 different ATG proteins have been identified in yeast, and their mammalian counterparts also have also been reported [5], [11]. Although the roles of a few ATG proteins in cancer have been characterized, the role of ATG10 is almost completely unknown. Here, we evaluated the relationship between ATG10 expression and clinicopathological features of sporadic colorectal carcinoma. Our findings show that increased ATG10 expression is strongly associated with lymph node metastasis and lymphovascular invasion in colorectal cancer.

## Results

### ATG10 Up-regulation in Colorectal Cancer

**Figure 1:**
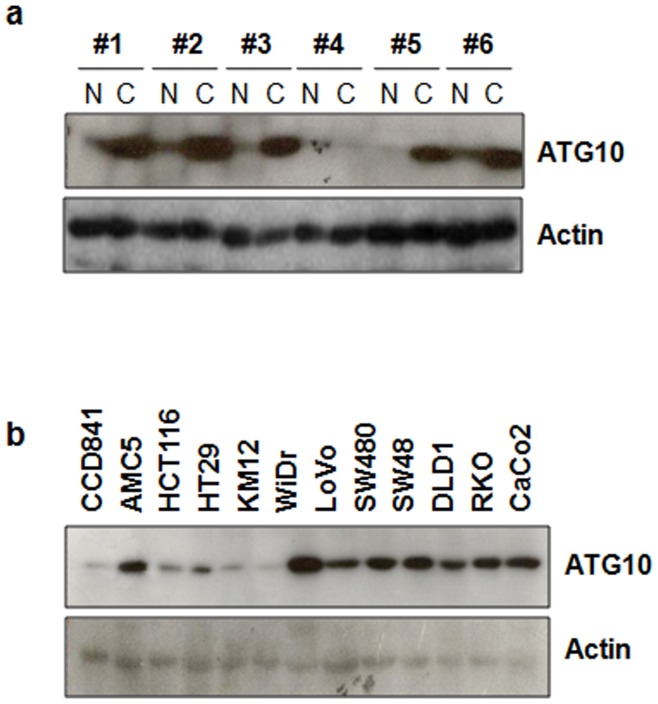
ATG10 expression is increased in colorectal cancer. (a) ATG10 expression in colorectal tissues was assessed by Western blot analysis (n = 37; T, tumor tissue; N, corresponding normal tissue). ATG10 expression was normalized to actin expression (n = 37). (b) Western blot analysis of ATG10 expression in several colorectal cell lines (Normal - CCD841; Cancer - HCT116, HT29, KM12C, WiDr, LoVo, SW480, SW48, HCT15, DLD1, RKO, CaCo2). ATG10 expression was quantified by densitometry.

To evaluate ATG10 expression in colorectal cancer, we first analyzed colorectal cancer tissues by Western blot. Tumor tissues and their surrounding normal tissues were obtained at the time of surgery. The result showed that ATG10 expression in tumors was significantly higher than that of the adjacent normal mucosa. ATG10 was increased in 18 of the 37 cases (48%) of colorectal cancer (Fig. 1a). We then examined ATG10 expression in colorectal cell lines. Consistent with the results obtained using tumor tissues, ATG10 expression was higher in cancer cell lines including AMC5, LoVo, SW480, SW48, HCT15, DLD1, RKO and CaCo2 than in the CCD841 normal colorectal cell line (Fig. 1b). Taken together, these results indicate that ATG10 is up-regulated in colorectal cancer.

### Relationship between ATG10 Expression and Tumor Progression and Invasion

**Figure 2:**
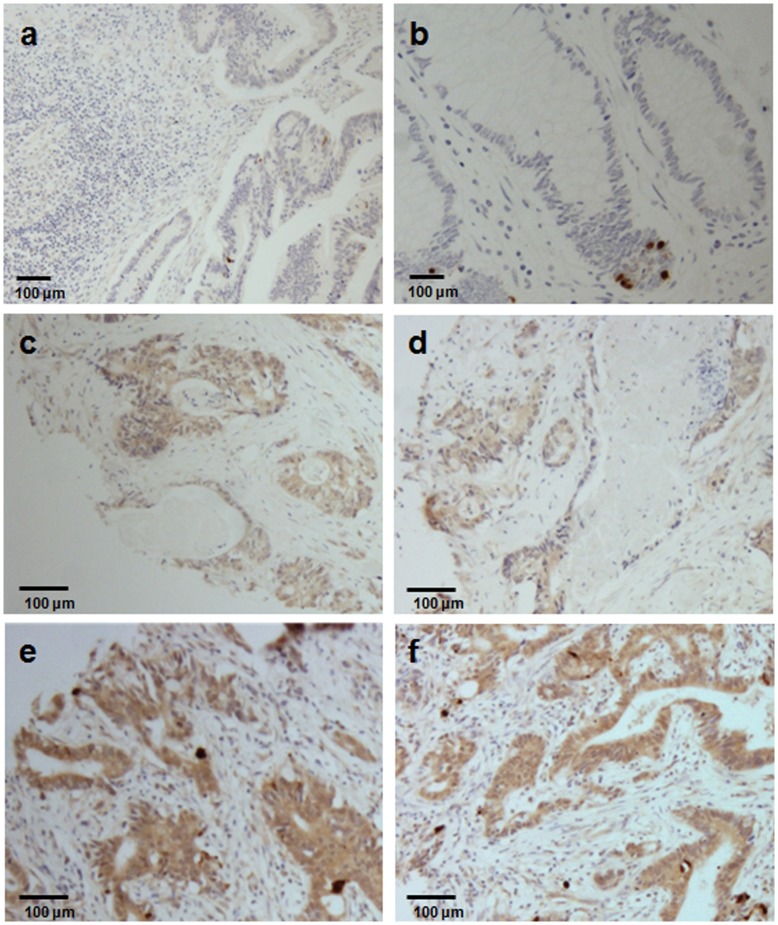
The expression pattern of ATG10 in colorectal cancer. The ATG10 expression pattern in colorectal cancer was determined by immunohistochemical analysis of a tissue microarray. (a and b) Negative specimens (−) with ≤10% staining (200), (c and d) specimens with 10% to 50% staining (+, ×200), and (e and f) specimens with >50% staining (+, ×200).

We further investigated the role of ATG10 in tumor progression and invasion. To examine clinicopathological features, immunohistochemical analysis of a tissue array with 127 colorectal cancer specimens was performed (Fig. 2 and Table 1). Tissues with no staining or weak staining (≤10%) were categorized as negative (−). Tissues with >10% staining were categorized as positive (+). Thirty of the 127 tumor specimens (24%) showed ATG10 expression. The relationship between ATG10 expression and clinicopathological features is shown in Table 2. The results showed that ATG10 expression was highly associated with lymphovascular invasion (*P*<0.001) and lymph node metastasis (*P* = 0.005). However, ATG10 expression was not significantly associated with age, sex, tumor site, serum carcinoembryonic antigen, or tumor proliferation. These results demonstrate that ATG10 expression is strongly associated with lymphovascular invasion in colorectal cancer.

**Table 1:** Clinicopathological features of 127 colorectal cancer patients.

Parameters	No. of patients
Male/female	59/68
Tumor site^a^, right/left/rectum	27/37/63
Growth type, expanding/infiltrative	103/24
AJCC stage^b^, I/II/III/IV	10/38/58/21
T category, 1/2/3/4	1/16/102/8
N category, 0/1/2	50/46/31
M category, 0/1	106/21
Grade, W/M/P	22/92/13
Lymphovascular invasion +	60
Patients with synchronous adenoma	30

Age, mean ± SD (58±12); Preoperative serum CEA, ng/ml, mean ± SD (47±379).

CEA, serum carcinoembryonic antigen; W/M/P, well-differentiated/moderately-differentiated/poorly-differentiated+mucinous. ^a^Right, cecum – splenic flexure of transverse colon; left, descending colon – sigmoid colon. ^b^Cancer staging according to the American Joint Committee on Cancer (7th ed., 2010).

**Table 2:** ATG protein expressions associated with clinicopathological feature (127 case).

Parameters	ATG10		
	# of (−) case (%)	# of (+) case (%)	*P*
Age, ≤/>50	48/49	11/19	0.295
Male/female	23/74	12/18	0.102
CEA, ≤/>6 ng/ml	65/32	20/10	1
Site, R/L+rectum	20/77	7/23	0.8
T^a^, 1+2/3+4	13/84	4/26	1
N^a^, 0/1+2	**45/52** **(46%/54%)**	**5/25** **(17%/83%)**	**0.005**
M^a^, 0/1	84/13	22/8	0.098
Growth, E/I	77/20	26/4	0.437
Grade, W+M/P	86/11	28/2	0.732
LVI, −/+	**64/33 (66/34)**	**3/27(10/90)**	**<0.001**
Adenoma, −/+	65/32	24/6	0.254
Recurrence, −/+	83/14	22/8	0.166

CEA, serum carcinoembryonic antigen;R/L, Right (cecum – splenic flexure of transverse colon)/left (descending colon – sigmoid colon);E/I, expanding/infiltrative; W/M/P, well-differentiated/moderately-differentiated/poorly-differentiated+mucinous; LVI, lymphovascular invasion. Bold font, p<0.05.

a Cancer staging according to the American Joint Committee on Cancer (7th ed., 2010).

### Relationship between ATG10 Expression and Patient Survival

**Figure 3:**
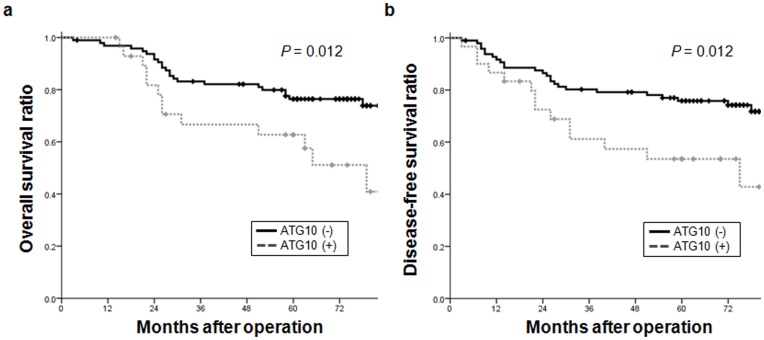
Relationship between ATG10 expression and survival of colorectal cancer patients. The relationship between ATG10 expression and 5-year disease-free survival (DFS) and overall survival (OS) rates of colorectal cancer patients were analyzed by Kaplan-Meier curves.

Results of the immunohistochemical analysis showed that ATG10 is closely associated with tumor invasion, which is known risk factor for recurrence and survival outcomes. Thus, we evaluated the relationship between ATG10 expression and the rates of 5-year disease-free survival (DFS) and overall survival (OS) in colorectal patients. The 5-year DFS and OS rates of patients bearing tumors that did not express ATG10 were significantly higher than those of patients bearing ATG10-expressing tumors (5-year OS [mean ± SEM], 76.4±0.4% *vs.* 57.5±0.1%, *P* = 0.012; 5-year DFS, 75.8±0% *vs.* 42.8±0.1%, *P* = 0.012) (Fig. 3). These results suggested that ATG10 may be a potential prognostic maker in colorectal cancer. In a multivariate analyses with potential survival variables, lymph node metastasis alone was significantly associated with survival parameters, whereas ATG10 expression was not (Hazard Ratio, 4.736 *vs.* 1.404; 95% Confidence Interval, 1.763–12.723 *vs.* 0.676–2.916; *P* = 0.002 *vs.* 0.363).

### Down-regulation of ATG10 Suppressed Cell Proliferation in Colorectal Cancer Cells

**Figure 4:**
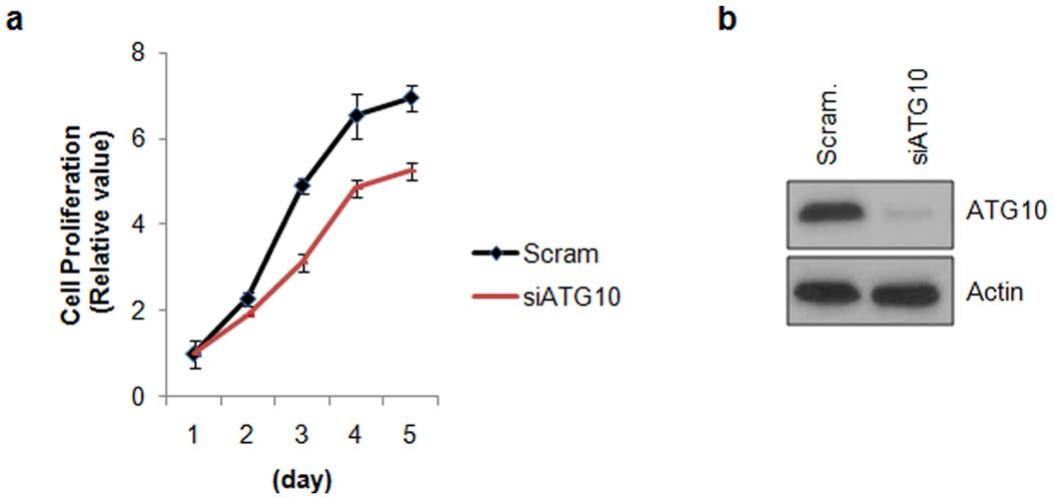
Silencing of ATG10 expression reduces cell proliferation in HCT116 cells. HCT116 cells were transiently transfected with either scrambled negative siRNA (Scram) or ATG10 specific siRNA (siATG10) then the cell proliferation rate was daily determined using a CCK-8 assay kit (a). The down-regulation of ATG10 by siRNA was confirmed with Western blot analysis (b). Data are represented by the mean±SEM (n = 3).

We further examined the effect of down-regulation of ATG10 on cell proliferation in HCT116 cells. Depletion of ATG10 expression by RNA interference slightly suppressed cell proliferation rate in HCT116 cells, suggesting that ATG10 has functional role in cell proliferation (Fig. 4).

## Discussion

Autophagy is controlled by ATG proteins and their regulators [5], [8]. ATG proteins initiate autophagosome formation through the autophagic conjugation system [2]. The role of ATG6 in cancer has been characterized. Previous studies have reported that decreased ATG6 expression promotes tumorigenesis in animal models, and ATG6 is down-regulated in various human cancers [12]–[15]. However, the role of other ATG proteins in tumorigenesis is not well understood.

In this study, we evaluated ATG10 expression in patients with sporadic colorectal cancer. ATG10 is an E2-like enzyme involved in Ub-like modification, which is essential for autophagosome formation. Previous studies have reported that several over-expressed Ub-E2 like proteins promote tumor development in various cancers [16]–[18]. However, the role of ATG10 in cancer has not yet been evaluated. Unlike ATG6, which is down-regulated in cancer, the present results indicate that ATG10 is increased in colorectal cancer. Furthermore, ATG10 expression was strongly associated with tumor invasion and metastasis. Our findings suggest that ATG10 may be an oncogenic protein. We also evaluated the effect of ATG10 over-expression on cell proliferation and in a mouse xenograft model using RKO carcinoma cells stably expressing ATG10. Cell proliferation and tumor growth were not significantly influenced by ectopic expression of ATG10 (data not shown). However, knock-down of ATG10 expression with siRNA suppressed cell proliferation rate in HCT116 cells. Thus, we are continuing to investigate the effect of up-regulated ATG10 on tumorigenesis.

Genetic alterations are responsible for the increased expression of many oncogenic proteins. The chromosomal region of ATG10 (5q14) is frequently lost in ovarian cancer, gastric cancer, and breast cancer [19], [20]. However, recent genome-wide DNA microarray studies have revealed that the 5q14 region is amplified in neurofibrosarcoma and pancreatic cancer [21], [22]. Copy number variations at 5q14 in colorectal cancer are not well understood. Thus future studies are needed to determine allelic alterations at this locus in colorectal cancer.

Autophagy seems to have dual functions in cancer. In particular, the timing of autophagy may be important for tumor development. In the early stage of tumorigenesis, autophagy appears to function as a tumor suppressor. As a result, inhibiting autophagy increases DNA damage, chromosomal alterations such as allelic loss and gain, and oxidative stress, which could lead to oncogenic events [23], [24]. However, autophagy also promotes tumorigenesis. Autophagy induction by detachment from the extracellular matrix assists tumor cell survival during anoikis, which contributes to dissemination and metastasis [25], [26]. Consistent with these previous studies, our results showed that ATG10 expression is closely associated with lymphovascular invasion and lymph node metastasis in colorectal cancer. These findings may be explained by the connection of tumor-replaced nodes or intravascular tumor aggregates with systemic lymph nodes via lymphovascular channels [27], [28]. Tumor invasion and metastasis are known risk factors for recurrence and survival outcome. According to this notion, we found that ATG10 expression in tumors was associated with lower survival rates. However, the relationship between ATG10 expression and clinical outcomes should be confirmed with a larger cohort to better understand the role of ATG10 in cancer.

In conclusion, ATG10 up-regulation in colorectal cancer is closely associated with lymphovascular invasion and lymph node metastasis, indicating that ATG10 may be useful as a prognostic marker and as a therapeutic target in colorectal cancer.

## Materials and Methods

### Patients and Tumor Specimens

To evaluate ATG10 expression, 37 colorectal cancer tissue specimens were randomly chosen from archival specimens from resections performed between June 1999 and May 2003 at the Asan Medical Center (Seoul, Korea). For the subsequent clinical validation of differential protein expression patterns, we evaluated tumor specimens from a total of 124 patients with sporadic colorectal cancer, including consecutive patients who underwent resection with curative intent (R0 resection, n = 119; R1 resection, n = 5) (Table 1). Patients with hereditary nonpolyposis colorectal cancer or familial adenomatous polyposis were excluded, as were those who underwent preoperative chemoradiation therapy. Recurrence, including regional and distant metastases, occurred in 22 of 124 patients (17.7%) who underwent curative resection during follow-up (mean, 58 months; range, 3–123 months). All patients provided written informed consent, and the study protocol was approved by the IRB (institutional review board), in accordance with the Declaration of Helsinki.

### Cell Lines and Reagents

CCD841 cells were kindly provided by Dr. S.Y. Rha (Yonsei University, Seoul, Korea) [29]. The AMC5 cell line was derived as previously described [30]. And other cancer cell lines such as HCT116, HT29, KM12C, WiDr, LoVo, SW480, SW48, HCT15, DLD1, RKO, and CaCo2 were purchased from ATCC (Manassas, VA). All cells were cultured at 37°C in a 5% CO_2_ incubator and maintained in RPMI1640 medium containing 10% fetal bovine serum and 1% penicillin/streptomycin (Invitrogen, Carlsbad, CA).

### Western Blot Analysis

Proteins from tissues and cells were prepared using protein sample buffer (62.5 mM Tris-HCl, 25% glycerol, 2% SDS, 5% 2-mercaptoethanol, 0.01% bromophenol blue) (BioRad, Hercules, CA). Proteins (approximately 50 ug) were quantitated by using the Bradford solution (BioRad) according to the manufacture’s instruction and then, separated by SDS-polyacrylamide gel electrophoresis and transferred to polyvinylidene fluoride membranes (BioRad). The membranes were incubated with primary antibodies against ATG10 (1∶3000; MBL, Nagoya, Japan) and actin (1∶10,000; Chemicon International, Temecula, CA). The membranes were then incubated with horseradish peroxidase-conjugated secondary antibodies (1∶5000; Pierce, Rockford, IL).

### Immunohistochemical Staining

Tissue array blocks were prepared using a precision instrument (Beecher Instruments, Sun Prairie, WI). Immunohistochemical staining based on the labeled streptavidin–biotin method was carried out using a Dako LSAB kit (Dako, Carpinteria, CA) with monoclonal ATG10 antibodies (MBL). Weak staining (≤10%) and no staining were scored as negative (−), and >10% staining was scored as positive (+). Specimens with negative immunochemical staining were examined twice to verify the results.

### Cell Proliferation Assay

Small interfering RNA for ATG10 (siATG10, siGENOME SMART pool) and scrambled control non-targeting siRNA (Scramble) were synthesized by Dharmacon, Inc. (Thermo Scientific, Chicago, IL). HCT116 cells were transfected with 0.5 µmol/L of siATG10 or scrambled siRNA with Lipofectamine 2000 (Invitrogen, Carlsbad, CA). Then the proliferation rate was measured using a cell proliferation assay kit (CCK-8) in accordance with the manufacturer’s protocol (Dojindo Corporation, Japan). Briefly, cells in 96-well plate were mixed with 10 µl of CCK-8 solution, and were then incubated for one hour in a CO_2_ incubator. The subsequent colorimetric change was measured using a Victor microtiter plate reader (PerkinElmer) set to monitor changes in absorbance at 450 nm.

### Statistical Analysis

Cross-table analysis using Fisher’s exact test with two-sided verification was used to compare immunohistochemical findings according to the clinicopathological features and recurrence incidence of patients. Primary endpoints were recurrence, 5-year DFS, and 5-year OS. Survival rates were compared by the Kaplan–Meier method with the log-rank test, and potent survival factors were verified using Cox’s regression model. The significance level of 5% was chosen for each analysis. Statistical analyses were conducted using SPSS software (version 19; SPSS Inc., Chicago, IL).
